# Pre- and Post- COVID-19 Pandemic Pneumonia Rates in Hospitalized Schizophrenia Patients

**DOI:** 10.3390/medicina61071251

**Published:** 2025-07-10

**Authors:** Ana-Aliana Miron, Petru Iulian Ifteni, Alexandra-Elena Lungu, Elena-Luiza Dragomirescu, Lorena Dima, Andreea Teodorescu

**Affiliations:** 1 Facultatea de Medicină, Universitatea Transilvania din Brașov, Bulevardul Eroilor 29, 500036 Brașov, Romania; aliana_mioc@yahoo.com (A.-A.M.); lorena.dima@unitbv.ro (L.D.); andre_martie@yahoo.com (A.T.); 2 Spitalul Clinic de Psihiatrie și Neurologie Brașov, Str. Prundului No. 7–9, 500123 Brașov, Romania; alexandraelenalungu@yahoo.com (A.-E.L.); luiza.dragomirescu@yahoo.com (E.-L.D.)

**Keywords:** schizophrenia, pneumonia, pandemic, polypharmacy

## Abstract

*Background and Objectives*: Schizophrenia is a disabling psychiatric condition, affecting around 1% of people worldwide. It has been ranked among the ten most disabling conditions globally. Alongside the psychological and social burdens imposed on individuals suffering from this disease, there are also serious complications regarding the physical health of these patients. Pneumonia is a significant cause of death in patients with schizophrenia. This group of patients also has a higher risk of developing pneumonia and all-cause mortality compared to those without schizophrenia, along with an increased overall mortality rate. A retrospective study revealed that advanced age, underweight, smoking, and the use of high-dose atypical antipsychotics increase the risk of pneumonia-related mortality in hospitalized patients. Our study aims to examine differences in factors associated with pneumonia in hospitalized patients with schizophrenia, before and after the COVID-19 pandemic, as well as to identify potential changes in clinical characteristics and outcomes. *Materials and Methods*: This is an observational, retrospective analysis, based on the review of medical records of psychiatric inpatients diagnosed with schizophrenia according to the DSM-5 criteria. Patients were selected according to the following criteria: both schizophrenia and pneumonia diagnoses, hospitalized in Spitalul Clinic de Psihiatrie si Neurologie Brasov during 1 March 2018–1 March 2020, and 1 March 2022–1 March 2024, respectively. *Results*: A total of 27 patients met the inclusion criteria; 13 patients (48%) were in the pre-pandemic group and 14 patients (52%) in the post-pandemic group. Contrary to other reports, our results showed relatively low pneumonia rates in hospitalized schizophrenia patients (1.02% pre-pandemic and 1.63% post-pandemic), and rates were higher in female patients (61.54% pre-pandemic and 71.43% post-pandemic). Post-pandemic, most cases (42.86%) were registered during summer, in a schizophrenia population with mostly urban residence and with lower smoking rates than the pre-pandemic group. Physical restraints were, however, more frequently utilized in the post-pandemic group. *Conclusions*: Pneumonia risk factors might register a change in the post-pandemic years. Polypharmacy and physical restraints are probably underestimated risk factors for pneumonia in schizophrenia patients, while a multidisciplinary approach and preventive measures might exert a protective role.

## 1. Introduction

Schizophrenia, a disabling psychiatric condition, is a multidimensional syndrome, affecting around 1% of people worldwide. It can exhibit a wide variety of psychotic, cognitive, mood, and motor manifestations. It is characterized by a triad of positive psychotic symptoms (such as delusions, hallucinations, or disorganized behaviors), negative manifestations (anhedonia, avolition, and blunted affect), and cognitive impairments (both executive function and mental processing speed may be affected). Schizophrenia ranks among the top 10 global disability causes [1,2].

Alongside the psychological and social burdens imposed on individuals suffering from this disease, there are also serious complications regarding the physical health of these patients. First and foremost, the life expectancy of these patients may be lowered compared to that of the general population, with a difference gap varying between 13 and 30 years [3]. A systematic review and meta-analysis conducted by Correll et al., investigating the mortality in schizophrenia patients, found that, among natural causes, pneumonia had the highest mortality risk (RR = 7.00, 95% CI: 6.79–7.23) [4]. Moreover, hospital-acquired pneumonia occurs more frequently in patients with schizophrenia, largely due to extended stays in closed wards, along with contributing factors like age, gender, and nutritional status [5].

Multiple risk factors for developing pneumonia in schizophrenia patients, both outpatients and inpatients, have been identified over time. Interestingly, among other more obvious risk factors (such as age, smoking, comorbidities), the specific treatment for schizophrenia has been identified by some authors as one of the important risk factors for pneumonia, particularly in hospitalized patients [5,6,7].

The prevalence of human immunodeficiency virus infection and infectious hepatitis is higher among patients with schizophrenia. Implementing interventions that have successfully reduced medical morbidity in the general population may help lower premature mortality in individuals with schizophrenia [8].

People with schizophrenia have a significantly higher risk of respiratory diseases, including pneumonia, COPD (chronic obstructive pulmonary disease), asthma, and tuberculosis, compared to the general population. This increased risk is influenced by high smoking rates, which negatively impact lung health, prenatal and childhood exposure, and poor socio-economic conditions with limited access to healthcare and adequate living conditions. Additionally, low physical activity levels and certain antipsychotics that promote weight gain can further affect respiratory function. There is also evidence that antipsychotics may increase the risk of acute respiratory failure in patients with COPD [9].

The long-term prognosis for patients diagnosed with schizophrenia is a highly variable factor, being dependent on the early detection of the disease, the pharmacological treatment, and the compliance of the patients with the prescribed treatment [10].

It is common for patients diagnosed with schizophrenia to experience a high relapse rate when treatment is discontinued. Moreover, a continuously administered treatment does not guarantee a complete lack of relapse [11].

Since the discovery of chlorpromazine in the 1950s, incredible advancements have been made in discovering new and more potent medications for treating patients afflicted with this disease. Nowadays, antipsychotics are largely classified into two large groups: the first generation of antipsychotics, also known as typical or conventional (chlorpromazine, haloperidol, loxapine, zuclopenthixol, etc.), and the second generation of antipsychotics, the so-called atypical ones (clozapine, olanzapine, quetiapine, risperidone, aripiprazole, etc.) [12]. Many atypical antipsychotics exert their effects primarily through 5-HT_2_A antagonism combined with D_2_ receptor antagonism or partial agonism, but they also interact with a range of other receptors, resulting in varied efficacy and side-effect profiles. The anticholinergic properties of certain antipsychotics, such as clozapine and olanzapine, contribute to xerostomia and impaired oropharyngeal coordination, thereby increasing the risk of aspiration events. Additionally, sedative effects mediated through histamine H_1_ and α_1_-adrenergic receptor antagonism can lead to a suppressed cough reflex and diminished mucociliary clearance, further predisposing individuals to respiratory infections [13,14,15,16]. These pharmacodynamic effects are often amplified in the context of antipsychotic polypharmacy due to cumulative receptor interactions across multiple neurochemical pathways [7]. One meta-analysis found antipsychotic use increased pneumonia incidence by almost 60% [17].

In 1952, chlorpromazine-induced symptoms resembling Parkinson’s disease were observed [18,19]. Typical antipsychotics are more susceptible to being the cause of extrapyramidal symptoms (EPSs) [20]. First-generation neuroleptics were associated with EPSs in 61.6% of patients according to a study of institutionalized patients with schizophrenia. Rates of EPSs are decidedly lower when using atypical antipsychotics, though the risk of EPSs increases with dose escalation [21]. This phenomenon seems to be due to the anticholinergic properties of atypical antipsychotics. Of the second generation of antipsychotics, the ones most often reported to have an increased anticholinergic effect are quetiapine, olanzapine, and clozapine [22].

Although not recommended in medical guides [23], trihexyphenidyl, a known anticholinergic medication, is often used as an adjunctive therapy in schizophrenia patients in a preventive manner to counteract the potential extrapyramidal effects caused by antipsychotics. As such, trihexyphenidyl, as another medication with a high anticholinergic mechanism of action, may prove to be a cause of a higher risk of developing pneumonia in patients being administered it [24].

It has been noticed that a higher anticholinergic effect may lead to a higher risk of pneumonia in patients, especially in the initiation phase of the treatment [25]. Anticholinergic medication is a class of drugs that block and inhibit the activity of acetylcholine, both in peripheral and in central nervous system synapses. The result of these actions leads to the inhibition of the parasympathetic nervous system due to the effect of the selective blockade of acetylcholine. Among the primary roles of the parasympathetic nervous system, we may count the involuntary actions of smooth muscle, primarily in the lungs and gastrointestinal tract [26].

The often encountered “dry mouth” anticholinergic side effect may cause mucosal damage, which, in turn, can be a serious risk factor for respiratory infection, due to the loss of the antimicrobial activity of saliva [27]. The dry mouth side effect is often the cause of oropharyngeal and esophageal swallowing impairments, which increase the risk for developing aspiration pneumonia [28]. Another possible risk factor for aspiration pneumonia is the acid reflux caused by conditions that affect esophageal motility [29]. Not in the least, the reduced flow of mucociliary transportation can increase bacterial stay in lungs, and thus the risk of respiratory infection [27].

As noted earlier, quetiapine, clozapine, and olanzapine are the atypical antipsychotics leaning more towards anticholinergic effects, with olanzapine also having a noticeably higher histaminic affinity, leading to metabolic side effects [30].

Additional drugs, often used in the long-term treatment of schizophrenia, are mood stabilizers and benzodiazepines, as well as hypnotic medication, of which the Z-drugs (e.g., zolpidem, zopiclone) represent a much-used subset. The last two classes of drugs may also contribute to a higher risk of pneumonia due to their ability to cause depression in the respiratory centers located in the brain, causing, in turn, sedation. Sedation may thus lead to pneumonia by increasing the risk of aspiration [31].

Aside from pharmacological therapy, physical restraint intervention, an often-controversial method, may also be used in the management of patients diagnosed with schizophrenia. Mechanical restraint is normally used with the purpose of restricting or subduing patients with pronounced psychomotor agitation [32]. Mechanical restraint is still considered necessary and continues to be used in many European countries [33]. Mechanical restraint has been reported to potentially have a negative impact on the short- and long-term evolution of patients, especially in the elderly population, with increased risks of nosocomial infections and pneumonia [34].

Pneumonia is a widespread acute respiratory infection that affects the alveoli and distal airways. Pneumonia is a frequently encountered cause of mortality and morbidity. It is classified into two main types: community-acquired and hospital-acquired pneumonia. A diverse range of microorganisms, including bacteria, respiratory viruses, and fungi, can be responsible for pneumonia, with their prevalence varying across different regions. The condition is more frequently observed in vulnerable populations, such as children under five and older adults with preexisting chronic illnesses. While pathogen characteristics contribute to the disease, its development is primarily influenced by the host’s immune response. Patients typically present with both respiratory and systemic symptoms, with diagnosis relying on clinical evaluation and radiological imaging. Timely identification of the causative pathogens is crucial, as delays or inadequate antimicrobial therapy can lead to poor outcomes. Advancements in antibiotic and non-antibiotic treatments, along with the development of rapid and precise diagnostic tools for detecting pathogens and antibiotic resistance, are essential for improving pneumonia management [35,36].

At the end of 2019, cases of pneumonia with an unknown cause emerged in Wuhan, China. Scientists later identified the responsible pathogen as a previously unknown beta coronavirus, now known as severe acute respiratory syndrome coronavirus 2 (SARS-CoV-2). Subsequently, the World Health Organization declared the disease caused by SARS-CoV-2, coronavirus disease 2019 (COVID-19), a pandemic. During the acute phase of COVID-19, the most common complications include pneumonia, respiratory failure, sepsis, and death. In addition to patient-specific factors, coinfections and superinfections play a crucial role in worsening the prognosis [37].

Following the outbreak of COVID-19, the proportion of non-COVID-19 viral pneumonia significantly declined, with the most notable decrease observed in Influenza A virus H1N1 pneumonia [38]. Moreover, a general decline in bacterial pneumonia cases was observed in 2020. This reduction may be attributed to decreased pathogen transmission due to social distancing measures and the use of personal protective equipment by healthcare personnel. However, microorganisms known to cause cavitating pneumonia, such as *Staphylococcus aureus* and *Klebsiella pneumoniae*, remained prevalent in 2020 [39].

## 2. Aims

Our study aims to examine differences in factors associated with pneumonia in patients with schizophrenia before and after the pandemic. We seek to identify potential changes in clinical characteristics and outcomes, providing insight into how the pandemic may have influenced the incidence, severity, and management of pneumonia in this vulnerable population.

## 3. Materials and Methods

### 3.1. Data Source

The study is an observational, retrospective analysis based on the review of medical records of psychiatric inpatients diagnosed with schizophrenia according to the DSM-5 criteria. All patients were admitted to the psychiatry departments of “Spitalul Clinic de Psihiatrie și Neurologie Brașov”. Our institution is a medical academic center in Brașov, România, with 150 beds for acute patients and 300 beds for long-term admission patients.

All patients who were included in the research signed an informed consent. The research has the Hospital’s Ethics Committee’s Approval (Approval no. 8/13 May 2025). Data were collected by board-certified psychiatrists from the paper and electronic files of the patients admitted to the hospital in the aforementioned period of time.

Extracted and analyzed data included sex, age, duration of illness, age of onset, date and duration of hospitalization, comorbidities, therapeutic management (antipsychotic type, formulation and dosage, mood stabilizer type and dosage, benzodiazepine type and dosage, hypnotic treatment, anticholinergic treatment, mechanical restraints), and transfers to other departments.

### 3.2. Study Design

All patients that were included in the analysis were admitted to the psychiatric department of Spitalul Clinic de Psihiatrie și Neurologie Brașov during the periods of 1 March 2018–1 March 2020 (the pre-pandemic group), and 1 March 2022–1 March 2024, respectively (the post-pandemic group). The period between March 2020 and March 2022 was excluded due to the COVID-19 pandemic, when the number of pneumonia cases was very high due to SARS-CoV-2 infections, and treatment protocols varied according to the available evidence at the time. Additionally, for the duration of the Emergency State during the pandemic, our hospital had to treat COVID-19 patients who were transferred from our Hospital’s Chronic Psychiatry departments; therefore, these data could not be taken into consideration, since the targeted population was not institutionalized patients. The study included all patients diagnosed with schizophrenia who developed pneumonia during their hospitalization. In all cases, the diagnosis of pneumonia was established based on the examination performed by the infectious disease specialist and/or internist, by correlating clinical findings (e.g., fever, tachycardia, tachypnea, and abnormal lung auscultation) and laboratory data (e.g., pulse-oxymetry, leucocyte count, neutrophil count, C reactive protein, and procalcitonin) [40]. All cases had imaging confirmation (chest X-ray or CT scan).

Patients diagnosed with schizoaffective disorder, as well as patients with multiple pneumonia episodes due to COPD, were excluded.

### 3.3. Statistical Analysis

After data collection, the information was entered into Microsoft Excel 2010. Statistical evaluation was performed using SPSS 20.00 (Statistical Package for the Social Sciences), with statistical significance accepted at a value of *p* < 0.05. The obtained results are expressed in numerical or percentage form.

To calculate the equivalent doses of chlorpromazine, we utilized the consensus method by Gardner et al. [41], Leucht et al.’s classical mean dose method [42], equivalence tables [43,44], and previous studies [45].

## 4. Results

### 4.1. General Findings

The total number of patients admitted to our hospital prior to the COVID-19 pandemic consisted of 5406 cases. Of these, a total of 1263 individuals were diagnosed with schizophrenia, and only 75 of the total number of admitted patients developed pneumonia during their stay in the hospital. Thirteen patients were diagnosed with both diseases during their admission in the hospital.

After the pandemic, the total number of patients admitted to our hospital decreased to 3960, of which 855 were diagnosed with schizophrenia. Fifty patients developed pneumonia during their hospitalization, and fourteen cases had in common both diagnoses.

Twenty-seven schizophrenia patients met the pre-established criteria for being admitted to the study, having been diagnosed with pneumonia during their stay in the hospital. Of these, 13 (48%) represent the pre-pandemic group, while the post-pandemic group consists of 14 patients (52%). Demographic characteristics of the two groups are detailed in Table 1.

Out of 27 patients, 9 patients (33.3%) were male, meaning that most (66.7%) of the schizophrenia patients who developed pneumonia were female, both pre- and post-pandemic. In the pre-pandemic period of the study, five patients (38.46%) were male, while after the pandemic, four patients (28.57%) were male.

**Figure 1:**
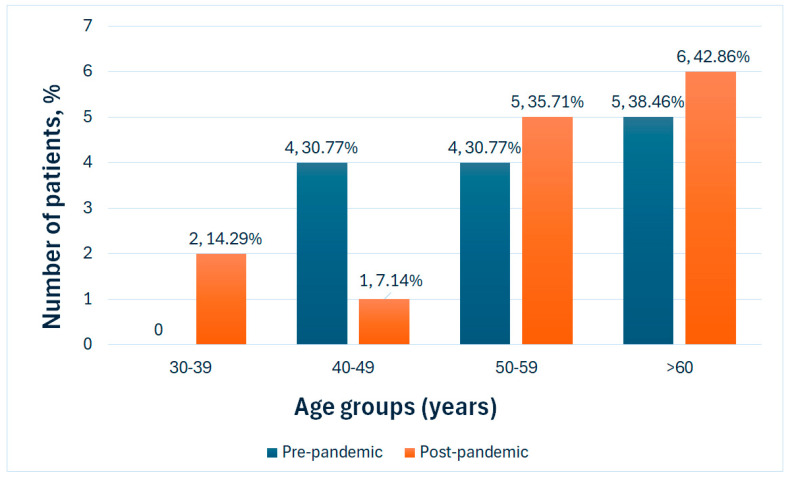
Age groups in the study population.

The mean age in the pre-pandemic sample was 54.85 (±10.05 SD), with the second sample of patients having a mean age of 57.07 (±12.5 SD) (Figure 1). The difference in the two samples of individuals does not have statistical significance (*p* = 0.6).

The mean age of onset in the pre-pandemic group was 29.77 years (±8.94 SD), while the post-pandemic mean age of onset was 29.43 years (±12.90 SD). No significant statistical difference was found (*p* = 0.9).

The mean duration of hospitalization was 30.00 days (±18.54 SD) in the pre-COVID-19 group and 49.36 days (±49.44 SD) in the post-pandemic group, with no statistically significant difference (*p* = 0.1).

The mean duration of illness (schizophrenia) was 25.08 years (±12.38 SD) in the pre-pandemic group and 27.64 years (±14.64 SD) in the post-pandemic group. No significant statistical difference was noted (*p* = 0.6).

In the pre-pandemic group, 9 individuals (69.23%) had an urban residence, while in the post-pandemic group, 13 individuals (92.86%) lived in an urban environment. One person (7.69%) had poor living conditions in the pre-pandemic sample, and similarly, one individual (7.14%) lived in poor conditions post-pandemic.

**Figure 2:**
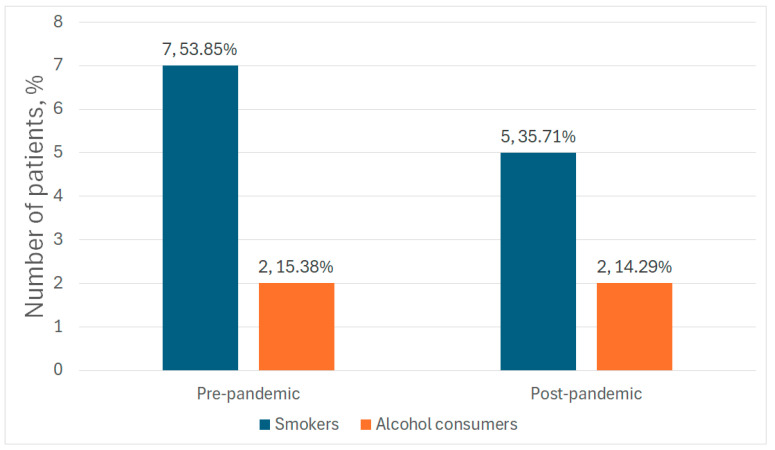
Nicotine and alcohol use rates in the study population.

In the pre-pandemic group, seven patients (53.85%) were smokers, and two (15.38%) were identified as alcohol consumers. In the post-pandemic group, there were five smokers (35.71%) and two individuals (14.29%) who reported alcohol use (Figure 2).

**Figure 3:**
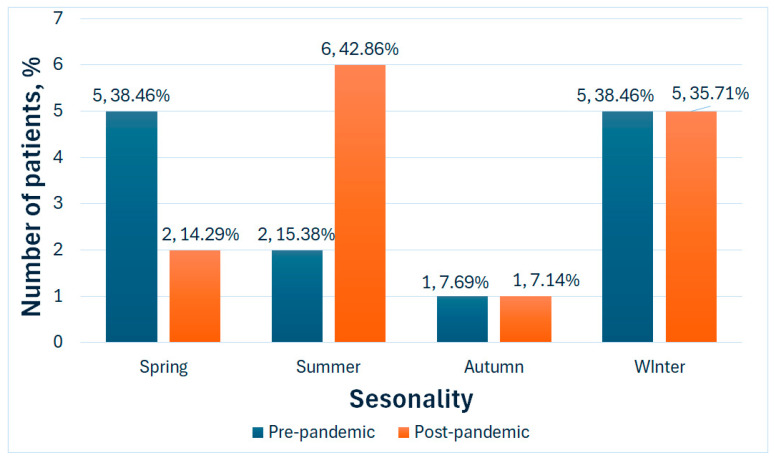
Seasonal trend in the study groups.

The seasonality of pneumonia cases in the pre-pandemic group showed a higher occurrence during spring and winter, with five cases (38.46%) in each season. In the post-pandemic group, the seasonal trend had a slightly different trend: only two cases (14.28%) occurred during spring, while six cases (42.86%) occurred during summer and five cases (35.71%) occurred during the winter season (Figure 3).

**Figure 4:**
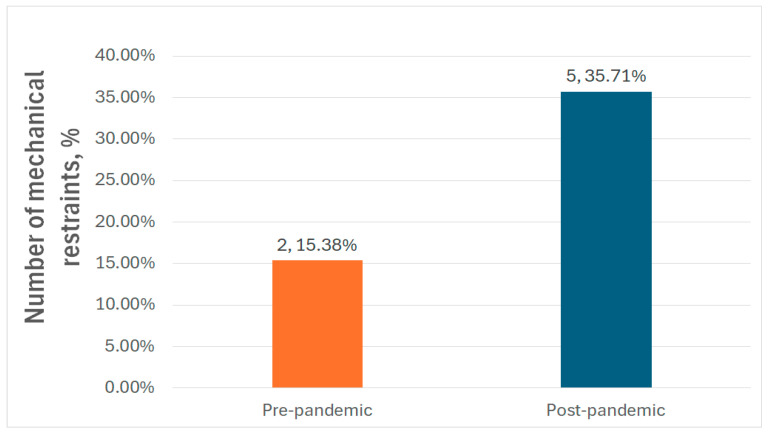
Mechanical restraint rates in the study groups.

We also be noted that, compared to the pre-pandemic period, where only 15.38% (2) of patients required mechanical restraint, in the post-pandemic group, this number increased to 35.71% (5), which is more than one-third of the total patients in the second period of our study (Figure 4).

**Figure 5:**
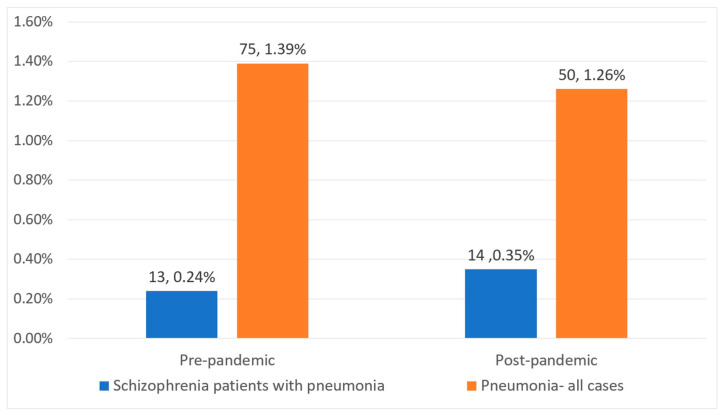
Pneumonia cases pre- and post-pandemic.

Globally, our results showed a decrease in the percentage of patients who developed pneumonia, regardless of the diagnoses, in the post-pandemic period (1.26%) compared to the pre-COVID-19 period (1.39%). However, among patients with schizophrenia, no significant changes were noted in the number of pneumonia cases (0.24% pre-pandemic versus 0.35% post-pandemic, *p* = 0.32) (Figure 5).

### 4.2. Findings Related to Antipsychotic Treatments

Antipsychotics used in the study groups are detailed in Table 2.

Most patients included in our study received oral antipsychotic medication, with long-acting injectables (LAIs) being used only in combination with oral antipsychotics (OAPs) as part of polypharmacy. Our findings show that, in the pre-pandemic period, the most commonly used antipsychotic was olanzapine (four cases, 30.77%), followed by clozapine (two individuals, 15.38%), and then risperidone and quetiapine, each administered to one patient (7.69%). Haloperidol was also administered to one patient (7.69%).

Polypharmacy appeared to be a common approach, with four patients in the pre-pandemic group receiving multiple antipsychotics. Among these, one patient was treated with a LAI (zuclopenthixol) in combination with haloperidol and quetiapine. Haloperidol was frequently used as part of combination therapy with various other medications.

In the post-pandemic period, risperidone and haloperidol were the most frequently prescribed antipsychotics, each used in three patients (21.43%). These were followed by olanzapine and quetiapine, each prescribed to two individuals (14.29%). Clozapine, when used as monotherapy, was administered to only one patient (7.14%). However, clozapine was more commonly found in polypharmacy regimens, with three patients (21.43%) receiving it in combination with other antipsychotics (Table 3).

**Figure 6:**
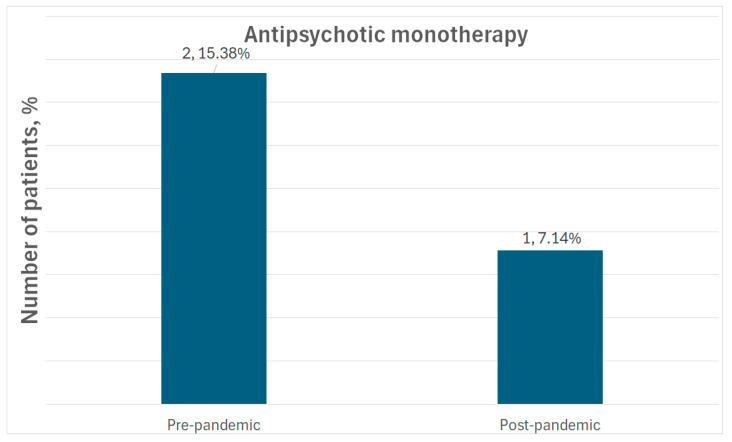
Antipsychotic monotherapy.

Our results show that antipsychotic monotherapy seems to be an exception rather than the rule, as seen in Figure 6.

### 4.3. Findings Related to Concomitant Medication

#### 4.3.1. Anticholinergic Treatment

Of the total 27 patients, 6 received concomitant anticholinergic treatment with trihexyphenidyl—3 patients (23.08%) in the pre-pandemic group and 3 (21.43%) in the post-pandemic group. The mean dose of trihexyphenidyl was 3.33 mg (±1.15 SD) in the pre-pandemic group and 4.66 mg (±1.15 SD) in the post-pandemic group. No significant statistical difference was found (*p* = 0.2).

#### 4.3.2. Benzodiazepines

Patients were also prescribed benzodiazepines (BZDs), specifically lorazepam and diazepam. In the pre-pandemic group, seven patients (53.85%) were administered lorazepam, with a mean dose of 3.2 mg (±1.78 SD). In the post-pandemic group, four patients (28.57%) received lorazepam, with a mean dose of 3.5 mg (±1.73 SD). There was no significant statistical difference (*p* = 0.7).

Diazepam was given to three patients (23.08%) in the pre-pandemic group, with a mean dose of 16.66 mg (±5.77 SD), while nine patients (64.29%) in the post-pandemic group received diazepam, with a mean dose of 19.44 mg (±10.14 SD). No significant statistical difference was observed (*p* = 0.6).

#### 4.3.3. Hypnotic Medication

Pre-pandemic, zopiclone was administered to 3 out of 13 patients (23.08%), with a mean dose of 7.5 mg (±0 SD). Post-pandemic, no patients were treated with zopiclone.

Zolpidem was given to two patients (15.38%) in the pre-pandemic group, with a mean dose of 10 mg (±0 SD). In the post-pandemic group, four patients (28.57%) received zolpidem, also at a mean dose of 10 mg (±0 SD).

#### 4.3.4. Mood Stabilizers

Valproate was the only mood stabilizer administered in both groups. In the pre-pandemic group, eight patients (61.54%) received valproate, with a mean dose of 837.5 mg (±333.57 SD). In the post-pandemic group, nine patients (64.29%) were treated with valproate, with a mean dose of 1055.55 mg (±283.33 SD). There was no significant statistical difference (*p* = 0.1).

Concomitant medication type and dose are detailed in Table 4.

#### 4.3.5. Antibiotics and Antiviral Medication

In the pre-pandemic group of our study, 11 out of 13 patients (84.62%) required antibiotic treatment for their pulmonary condition, suggesting that bacterial infections were the primary cause. In the post-pandemic period, when viral agents were responsible for many pneumonia cases, antiviral medication was also used in the treatment of two patients (14.29%). One of these cases also required antibiotic treatment. Still, 13 out of 14 patients (92.86%) in the post-pandemic group received antibiotic treatment (Table 5).

### 4.4. Complications

**Figure 7:**
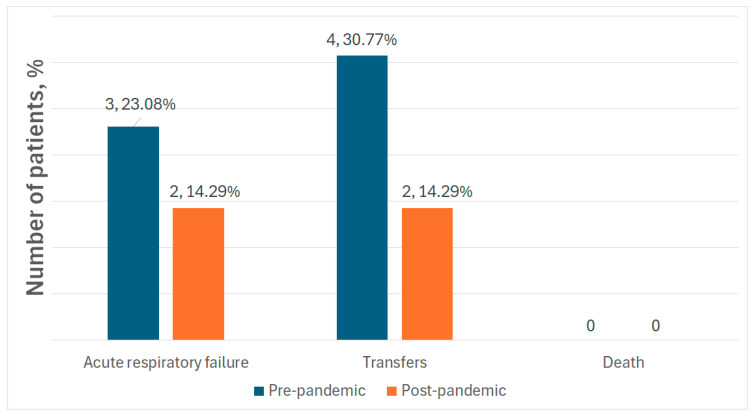
Pneumonia-related complications.

Concerning the complications related to health conditions secondary to pneumonia, our data demonstrate significant improvements in the incidence of acute respiratory failure and the need for patient transfers in the post-pandemic group. Additionally, mortality rates have remained at zero across all periods analyzed (Figure 7).

## 5. Discussion

Our research compared various clinical and demographic parameters of psychiatric patients diagnosed with schizophrenia and who developed pneumonia while being admitted, before and after the COVID-19 pandemic. This analysis provides an overview of changes in potential risk factors for pneumonia in schizophrenia patients, namely demographics, hospitalization duration, socioeconomic factors, seasonal trends, comorbidities, and medication pre- and post-pandemic.

Pneumonia is an important cause of death in patients with schizophrenia. This group of patients also has a higher risk of developing pneumonia and all-cause mortality compared to those without schizophrenia, along with an increased overall mortality rate.

In opposition to other previous reports, our results showed surprisingly low pneumonia rates in hospitalized patients: pre-pandemic, pneumonia rates were 1.38% among inpatients regardless of diagnosis, and 1.02% among patients with schizophrenia; post-pandemic, pneumonia rates were similarly low: 1.26% among all-diagnosis inpatients, and 1.63% among schizophrenia patients.

Earlier research has identified a high rate of hospital-acquired pneumonia (HAP) among Chinese patients with mental disorders. For instance, a large psychiatric hospital in Sichuan reported a 7.8% incidence of HAP in middle-aged and elderly patients (aged 50 and above) with schizophrenia [46]. Similarly, specialized medical institutions in Taiwan recorded an incidence rate of 14.7 cases per 1000 individuals per year among patients with severe mental disorders [47].

Our finding is clinically significant in several ways. The lower incidence compared to previous studies may reflect the effectiveness of institutional infection prevention protocols, particularly those reinforced during and after the COVID-19 pandemic. The presence of multidisciplinary oversight (internal medicine specialist, infectious disease specialist, epidemiology department, and strict regulations of antibiotics and antivirals) likely contributed to early detection, timely intervention, and mitigation of risk factors (e.g., aspiration, poor hygiene, and prolonged sedation) that are common in psychiatric inpatient settings. The change in the patients’ profile in the post-pandemic group suggests that new or evolving risk factors may be at play in the post-pandemic era, possibly related to differential impacts of COVID-19, medication regimens, or psychosocial stressors. Finally, we interpret the low incidence as a positive indicator of institutional control but also recognize the importance of continued surveillance, especially for emerging atypical patterns, such as the gender-related findings reported here.

Our results showed that pneumonia was more likely to appear in female patients, both in the pre-pandemic group (61.54%) and in the post-pandemic group (71.43%). This finding is opposed to previous reports; for instance, Yang et al. found that the incidence of pneumonia is significantly higher in male schizophrenia patients, most likely due to long-term smoking habits and poorer oral hygiene [46]. The unexpected rise in pneumonia among female schizophrenia patients after the pandemic likely reflects a synergistic impact of COVID-19’s long-term health effects, psychiatric and medical comorbidities, differential healthcare disruption, and medication effects by gender, and institutional care dynamics [48]. Post-pandemic shifts in immune vulnerability might also explain these results: females may have experienced more lingering effects from COVID-19, such as chronic inflammation or immune dysregulation, which could increase susceptibility to respiratory infections like pneumonia. Some evidence suggests that autoimmune and post-viral syndromes are more common in women, potentially increasing the risk of atypical respiratory complications after the pandemic [49].

Also, some studies suggest that females with schizophrenia may be prescribed higher cumulative doses of antipsychotics or may be more likely to experience side effects such as sedation and dysphagia, which increase the risk of aspiration pneumonia [50]. Medications such as clozapine have been linked to increased pneumonia risk, and gender differences in metabolism or adherence could play a role post-pandemic [51].

This highlights the importance of gender-sensitive healthcare monitoring in psychiatric populations, especially in post-pandemic recovery. Future studies should explore this trend prospectively and across multiple centers to validate and understand its implications.

Contrary to other reports, we noted an interesting shift in seasonal occurrence of pneumonia: while pre-pandemic, most cases were registered in spring and winter months, in the post-pandemic group, most cases (42.86%) were reported during the summer season. Usually, pneumonias register a peak of incidence in winter [52]; our finding might suggest that entirely different environmental or behavioral factors contribute to the development of pneumonias in the post-pandemic group. Indeed, our results showed a lower percentage of smokers in the post-pandemic group and a higher rate in the urban-based population, but also a higher rate of physical restraints and polypharmacy.

A 24-month multi-center observational study involving 602 patients with schizophrenia-related psychoses found that individuals with severe mental illness (SMI) experienced significantly more respiratory symptoms and poorer lung function compared to the general population. Conducted across six UK sites, the study highlighted that these respiratory issues were closely linked to lifestyle factors, with obesity, particularly among younger patients, playing a key role in the elevated risk [53].

A retrospective study shows that older age, underweight, smoking, and the use of atypical antipsychotics, especially in high doses, increase the risk of pneumonia-related mortality in hospitalized patients. It is recommended to minimize the doses and types of antipsychotics while maintaining the psychiatric stability of the patients [54,55]. Recent research by Chen et al. noted that the key risk factors for hospital-acquired pneumonia in individuals with schizophrenia include the use of benzodiazepines (particularly clozapine), combinations of antipsychotics, mood stabilizers, modified electroconvulsive therapy (MECT), longer hospital stays, existing health conditions, high blood sugar levels, and issues like excessive saliva or difficulty swallowing. Additionally, older age, smoking, alcohol consumption, malnutrition, and pre-existing illnesses further increase the risk [5]. Our results showed similar age of onset and duration of illness, and no significant difference in patients’ age in the pre- and post-pandemic groups, with a slight reduction in smoking rates in the post-pandemic group. This particular change in smoking habits could be linked to increased health awareness, while the stability in alcohol consumption suggests that substance use behaviors remained relatively unchanged.

Our findings suggest notable shifts in psychiatric patient profiles and associated health conditions. A significant increase in the duration of hospitalization post-pandemic may indicate a greater severity of illness or delayed access to care. Additionally, a rise in hepatopathy and stroke cases post-pandemic suggests potential long-term health consequences of the pandemic.

The increase in mechanical restraints that we found in the post-pandemic group (35.71% post-pandemic versus 15.38% pre-pandemic) raises concerns about the potential impact of the pandemic on schizophrenia patients’ behavioral symptoms or agitation levels. Also, the higher rate of mechanical restraints could be a possible explanation for the shift we noted in pneumonia patient characteristics. It has been previously reported that the use of physical restraints in psychiatric inpatients is a significant risk factor for aspiration pneumonia and thromboembolic events [56]. We also noted that, in the post-pandemic group, the mean duration of illness and the mean duration of hospitalization were higher than in the pre-pandemic group, suggesting the emergence of more severe and complex psychiatric presentations, possibly linked to delayed access to care, greater psychosocial stressors, or a higher threshold for hospitalization during the pandemic. Other authors have also noted that in the post-pandemic years, admissions related to externalizing symptoms—such as aggression, risk of harm to others, and substance use disorders—were consistently higher compared to the same period in the five preceding years. In contrast, admissions for internalizing symptoms like social withdrawal and depression remained consistently lower [57]. Additionally, the lack of certain antipsychotics that have been previously proven effective in controlling acute schizophrenia symptoms, such as loxapine, zuclopenthixol acetate, or olanzapine and aripiprazole injectable formulations, may have contributed to the increase in the use of mechanical restraints. There has been no change in the institutional policy regarding mechanical restraints.

Regarding the role of antipsychotics, our study found that before the pandemic, olanzapine was the most commonly used antipsychotic, followed by clozapine and then risperidone and quetiapine. Post-pandemic, risperidone and haloperidol were the most commonly used antipsychotics, followed by olanzapine and quetiapine, with clozapine being the least utilized. The shift in antipsychotic usage from the pre-pandemic to the post-pandemic period suggests potential changes in prescribing patterns, patient needs, or clinical guidelines. However, in the post-pandemic period, there was a noticeable shift toward more frequent use of clozapine in polypharmacy regimens, particularly among patients receiving multiple antipsychotics. This trend may suggest an increasing dependence on clozapine for managing treatment-resistant cases. The increase in polypharmacy, in the use of antipsychotic associations, and in the chronic use of concomitant medications [58] may be partly responsible for pneumonia cases in a different type of patient post-pandemic [5,46].

We noted that, in the post-pandemic group, besides the change in the antipsychotics prescribed, there was also an increase in the antipsychotic dosage used for most antipsychotics. As previously noted, our data suggest that in the post-pandemic group, patients might have had more severe episodes of schizophrenia (longer duration of illness, longer hospitalizations), which might also explain the necessity for higher doses of antipsychotics or more incisive types of antipsychotics, such as haloperidol.

A large Finnish cohort study involving 61,889 individuals diagnosed with schizophrenia or schizoaffective disorder highlighted several key risk factors for pneumonia. Over the follow-up period (1996–2017), 14.4% of patients were hospitalized for pneumonia, with a 30-day mortality rate of 12.8%. The study identified age as a significant factor, with pneumonia risk doubling every five years after age 50. Male sex, especially in those over 40, was also linked to a higher risk. In terms of antipsychotic treatment, monotherapy was associated with a greater pneumonia risk, whereas polytherapy did not increase the risk, possibly due to the more balanced receptor targeting, which may reduce excessive cholinergic stimulation. Specific antipsychotics were identified as high-risk when prescribed at higher doses: quetiapine (>440 mg/day), clozapine (>180 mg/day), and olanzapine (>11 mg/day). In contrast, first-generation antipsychotics were not linked to an increased risk. Mechanistically, the anticholinergic effects of these drugs may contribute to esophageal dysfunction and excessive sedation, increasing the risk of aspiration pneumonia. Antihistaminergic properties may further compound this risk by deepening sedation [22].

Interestingly, our results showed a reduction in pneumonia complications, such as acute respiratory failure (14.29% post-pandemic, versus 23.08% pre-pandemic), and in patients’ transfers to general hospitals due to pneumonia complications (14.29% post-pandemic, versus 30.77% pre-pandemic). No deaths were reported in our study, and no particular associations with a certain bacterial or viral species were found.

These findings might also raise a question regarding the potential protective role of antipsychotic treatment; this was already suggested by Moga et al. regarding the protection from severe manifestations of COVID-19 and from a detrimental course of illness in antipsychotic-treated schizophrenia patients [59].

Regarding clozapine treatment, existing data are inconsistent. Although clozapine may be the most efficacious antipsychotic and the best for treatment-refractory schizophrenia, pneumonia seems to be among the greatest causes of mortality in clozapine-treated patients [60]. However, a meta-analysis reported significantly lower deaths in patients continuously treated with clozapine compared to other antipsychotics [61]. Our study did not show an association between clozapine treatment and pneumonia.

A recent Finnish study [62] found that pneumonia and severe gastrointestinal complications are more common with the use of clozapine than previously believed. The study analyzed data over a period of up to 25 years, involving 2659 patients diagnosed with schizophrenia who were treated with clozapine, to estimate the real burden of clozapine adverse events (ADE).

Up to 30% of patients with schizophrenia treated with clozapine developed pneumonia, and 5% experienced ileus within 20 years of starting the treatment. Both events were significantly associated with increased mortality among clozapine users (ileus: OR = 4.5; pneumonia: OR = 2.8). Genetic tests could help identify patients at increased risk of clozapine-induced pneumonia, observed in two genes involved in clozapine metabolism. Reduced activities of CYP2C19 and CYP1A2 were associated with a higher risk of pneumonia. Thus, clozapine-induced ileus and pneumonia were significantly more frequent than previously reported and were associated with increased mortality [62].

The existing literature underscores the heightened risk of pneumonia and other respiratory conditions in schizophrenia patients, exacerbated by smoking, metabolic side effects of antipsychotics, and socioeconomic disparities. Notably, pneumonia remains a leading cause of mortality in clozapine-treated patients, emphasizing the necessity for vigilant monitoring and early intervention strategies [51].

Anticholinergic medication may elevate the risk of pneumonia in elderly adults, particularly during the initial phase of treatment. This risk is further heightened when older patients are prescribed high-potency anticholinergic drugs [25]. Furthermore, medication that acts as an agonist of benzodiazepine receptors (including benzodiazepines and z-drugs) increases the risk of dose-dependent pneumonia requiring hospitalization, especially in the case of midazolam [63]. Supporting these data, our results showed an increase in benzodiazepine use as an adjunctive treatment in the post-pandemic period (92.86% post-pandemic vs. 69.23% pre-pandemic), and in concomitant use of mood stabilizers plus benzodiazepines (64.29% post-pandemic vs. 46.15% pre-pandemic), while anticholinergic use registered a slight drop (21.43% post-pandemic vs. 23.08% pre-pandemic).

Our findings pointed to a slight decrease in the percentage of patients who developed pneumonia during the post-pandemic period (1.38%), regardless of psychiatric diagnosis, compared to the pre-COVID-19 period (1.02%). This reduction can be attributed to several protective factors that emerged as a result of changes in patient behavior, hospital policies, and medical management during and after the pandemic.

One of the most notable protective factors was a decrease in smoking among the post-pandemic patient group. This can be explained by the fact that, due to stricter hospital regulations, patients remained within the hospital premises throughout their stay and were only allowed to leave the building upon discharge. As a result, their exposure to smoking-related respiratory risks was significantly reduced. The reduced smoking noted during the post-pandemic period was not merely hypothesized but reflected an institutional change aligned with national policy. Post-pandemic, our hospital enforced a strict no-smoking policy, including in psychiatric wards; this is notified to all patients upon admission, and via visible signage and disciplinary procedures overseen by internal regulators and the epidemiology department. Hygiene improvements were supported by internal protocols and monitored by the hospital’s epidemiology department. Furthermore, these patients were less exposed to adverse weather conditions, such as cold temperatures and humidity, which are known to increase the risk of respiratory infections, including pneumonia [5,55].

Another key factor that may have contributed to the decline in pneumonia cases was the improvement in early detection and timely intervention. Routine testing protocols implemented during the pandemic allowed for the early identification of infections, leading to prompt treatment and better outcomes. Additionally, our hospital benefited from the presence of internal medicine and infectious disease specialists, ensuring that patients received specialized care when needed. This multidisciplinary approach likely played a crucial role in preventing complications and reducing the overall pneumonia incidence.

Moreover, antibiotic administration was more strictly regulated during and after the post-pandemic period. The use of antimicrobial agents followed well-defined therapeutic protocols, ensuring that antibiotics were prescribed only when necessary and in accordance with evidence-based guidelines. This controlled approach may have helped in preventing inappropriate antibiotic use, reducing the risk of bacterial resistance, and improving overall treatment efficacy.

Despite this, pneumonia cases among patients with schizophrenia did not show a significant reduction in incidence. One possible explanation for this finding is the inherent vulnerability associated with schizophrenia. Patients with schizophrenia often have multiple risk factors that predispose them to respiratory infections, including impaired self-care, cognitive dysfunction, and a higher likelihood of comorbid conditions. Additionally, the pharmacological treatment of schizophrenia, particularly the use of antipsychotic medications with sedative and anticholinergic properties, may contribute to an increased risk of pneumonia by affecting respiratory function, swallowing reflexes, and immune responses.

Another aspect to be considered is the possible confounding influence of improved oral and respiratory hygiene in the post-pandemic era. Enhanced public health behaviors such as increased mask usage and hand hygiene may have contributed to a general decline in respiratory infections, including pneumonia, in the broader population [64]. However, our study population consisted of individuals with acute schizophrenia, a clinical group known to exhibit limited adherence to hygiene-related public health measures, especially during acute psychotic episodes. As reported in prior studies, such patients frequently demonstrate reduced insight, impaired judgment, and behavioral disorganization, which may significantly limit compliance with mask-wearing, regular handwashing, or other preventive behaviors [65].

Given these well-documented challenges in hygiene compliance among acutely ill psychiatric inpatients, we believe that the influence of improved population-level hygiene practices post-COVID-19 may be less applicable or measurable within our specific cohort. Hence, general improvements in community hygiene may not translate effectively to high-risk psychiatric populations. Furthermore, there are no standardized metrics currently available to quantitatively assess individual-level hygiene compliance in this population, making statistical adjustment for this factor unfeasible.

We emphasize that awareness of pneumonia risk represents an important preventive factor. Measures such as smoking reduction, environmental temperature regulation, limited use of air conditioning, and avoidance of direct exposure to cold, rain, or snow have contributed to the reduction in pneumonia cases. We therefore consider the occurrence of pneumonia to be primarily a consequence of illness-related harmful behaviors, unmet basic needs, and insufficient implementation of preventive strategies in other hospital settings.

### Strengths and Limitations

The major strength in our research resides in reflecting the real-life comorbidity of schizophrenia and pneumonia, before and after the COVID-19 pandemic. It also highlights possible associations of pneumonia in schizophrenia patients, with complex psychotropic and concomitant treatment. Furthermore, our research highlights valuable protective factors, which might be taken into consideration for future research.

While our study provides valuable insights, several limitations must be acknowledged. First, the sample size may not be large enough to generalize findings to broader populations. Second, as it is an observational study, it makes it difficult to establish direct causal relationships between the pandemic and changes in patient characteristics, medication use, or hospitalization trends. To confirm and expand the findings, given the retrospective nature and small sample size of the current study, we are considering conducting a prospective study to minimize bias and allow for better control of confounding variables, including a larger population to improve the statistical power and generalizability of the results, and expanding the study to multiple centers to validate findings across different settings and patient populations.

Another limitation is the potential impact of unmeasured confounding factors, such as healthcare accessibility, which may have influenced the observed trends. Furthermore, retrospective data collection may introduce bias due to inconsistencies in medical record documentation.

Medication adherence and patient-specific factors, such as lifestyle changes and social support, were not extensively analyzed, limiting the ability to determine their role in post-pandemic shifts. Additionally, the study does not account for variations in clinical practice across different healthcare institutions, which may influence prescribing patterns and hospitalization criteria.

## 6. Conclusions

Pneumonia risk factors in schizophrenia inpatients might be different in the post-pandemic years compared to what was classically known. Polypharmacy and physical restraints may have a bigger role in the pathogenesis of pneumonia in schizophrenia patients.

Better hygiene and control of intra-hospital infections, as well as more judicious antibiotics administration, may lead to a decrease in the rate of pneumonia in hospitalized schizophrenia patients. A multidisciplinary approach to hospitalized schizophrenia patients may also exert a protective role for pneumonia.

Future research should focus on larger, multi-center studies with prospective designs to better assess long-term outcomes and refine strategies for improving psychiatric care in response to global health crises.

## Figures and Tables

**Table 1:** Demographic and clinical characteristics.

Parameters	Pre-Pandemic	Post-Pandemic	*p*-Value
Total patients (N, %)	13 (48%)	14 (52%)	0.8
Male gender (N, %)	5 (38.46%)	4 (28.57%)	0.5
Mean age (mean, ± SD)	54.85 (±10.05)	57.07 (±12.5)	0.6
Age of onset (mean, ± SD)	29.77 (±8.94)	29.43 (±12.9)	0.9
Duration of hospitalization (mean, ± SD)	30 (±18.54)	49.36 (±49.44)	0.1
Duration of illness (mean, ± SD)	25.08 (±12.38)	27.64 (±14.64)	0.6
Urban residence (N, %)	9 (69.23%)	13 (92.86%)	0.1
Poor living conditions (N, %)	1 (7.69%)	1 (7.14%)	0.9
Smokers (N, %)	7 (53.85%)	5 (35.71%)	0.3
Alcohol consumers (N, %)	2 (15.38%)	2 (14.29%)	0.9
Mechanical restraint (N, %)	2 (15.38%)	5 (35.71%)	0.2

**Table 2:** Antipsychotic types and doses.

AP (Type, Formulation)		No Cases (%)	Mean Dose (mg; ±SD)	CPZ Equivalent (mg)	*p*-Value
Quetiapine	Pre-pandemic	1 (7.69%)	600	800	0.5
	Post-pandemic	2 (14.29%)	600 (±282.84)	800	
Olanzapine	Pre-pandemic	4 (30.77%)	13.75 (±4.79)	275	0.3
	Post-pandemic	2 (14.29%)	15	300	
Haloperidol	Pre-pandemic	1 (7.69%)	5	250	0.3
	Post-pandemic	3 (21.43%)	5.66 (±4.04)	283	
Clozapine	Pre-pandemic	2 (15.38%)	325 (±318.2)	165.5	0.5
	Post-pandemic	1 (7.14%)	400	200	
Risperidone	Pre-pandemic	1 (7.69%)	6	300	0.3
	Post-pandemic	3 (21.43%)	3.5 (±0.86 SD)	175	
Polypharmacy	Pre-pandemic	4 (30.77%)	-	-	-
	Post-pandemic	3 (21.43%)	-	-	

**Table 3:** Antipsychotic associations.

Antipsychotics Used	Pre-Pandemic (N, %)	Post-Pandemic (N, %)
HAL + ZUC + QUE	1 (7.69%)	-
HAL + QUE	1 (7.69%)	-
HAL + RIS	1 (7.69%)	-
HAL + OLZ	1 (7.69%)	-
CLZ + RIS	-	1 (7.14%)
CLZ + FLX	-	1 (7.14%)
CLZ + ARI	-	1 (7.14%)

Legend: OLZ = olanzapine; RIS = risperidone; ARI = aripiprazole; QUE = quetiapine; HAL = haloperidol; FLX = flupenthixol; ZUC = zuclopenthixol; CLZ = clozapine.

**Table 4:** Concomitant medication.

Medication Type			Number of Cases (%)	Mean Dose (mg; ±SD)	*p*-Value
Anticholinergic (Trihexyphenidyl)		Pre-pandemic	3 (23.08%)	3.33 (±1.15)	0.2
		Post-pandemic	3 (21.43%)	4.66 (±1.15)	
BZD	Lorazepam	Pre-pandemic	7 (53.85%)	3.2 (±1.78)	0.7
		Post-pandemic	4 (28.57%)	3.5 (±1.73)	
	Diazepam	Pre-pandemic	3 (23.08%)	16.66 (±5.77)	0.6
		Post-pandemic	9 (64.29%)	19.44 (±10.14)	
Hypnotic medication	Zopiclone	Pre-pandemic	3 (23.08%)	7.5	-
		Post-pandemic	0	0	
	Zolpidem	Pre-pandemic	2 (15.38%)	10	0.4
		Post-pandemic	4 (28.57%)	10	
Mood stabilizers (Valproate)		Pre-pandemic	8 (61.54%)	837.5 (±333.57)	0.1
		Post-pandemic	9 (64.29%)	1055.55 (±283.33)	

**Table 5:** Antibiotic and antiviral treatments.

Medication Type			Number of Cases (N, %)
Antibiotics	Cefuroxime	Pre-pandemic	1 (7.69%)
		Post-pandemic	1 (7.14%)
	Ceftriaxone	Pre-pandemic	4 (30.76%)
		Post-pandemic	1 (7.14%)
	Amoxicillin + Clavulanic Acid	Pre-pandemic	1 (7.69%)
		Post-pandemic	1 (7.14%)
	Ampicillin + Sulbactam	Pre-pandemic	0
		Post-pandemic	1 (7.14%)
	Levofloxacin	Pre-pandemic	1 (7.69%)
		Post-pandemic	3 (21.42%)
	Sultamicillin	Pre-pandemic	1 (7.69%)
		Post-pandemic	0
	Ceftriaxone + Levofloxacin	Pre-pandemic	2 (15.38%)
		Post-pandemic	0
	Cefalexin + Sultamicillin	Pre-pandemic	1 (7.69%)
		Post-pandemic	0
	Azithromycin	Pre-pandemic	0
		Post-pandemic	2 (14.28%)
	Meropenem + Vancomycin	Pre-pandemic	0
		Post-pandemic	1 (7.14%)
	Meropenem	Pre-pandemic	0
		Post-pandemic	2 (14.28%)
	Ceftazidime	Pre-pandemic	0
		Post-pandemic	1 (7.14%)
Antiviral medication	Favipiravir	Pre-pandemic	0
		Post-pandemic	1 (7.14%)
	Favipiravir + Remdesivir	Pre-pandemic	0
		Post-pandemic	1 (7.14%)

## Data Availability

Data were retrieved from the paper and electronic documents of the patients. The datasets used and/or analyzed during the current study are available from the corresponding author upon reasonable request.

## Abbreviations

The following abbreviations are used in this manuscript:

COVID-19	Coronavirus Disease 2019
DSM-5	Diagnostic and Statistical Manual of Mental Disorders, Fifth Edition
EPS	Extrapyramidal symptom
SARS-CoV-2	Severe Acute Respiratory Syndrome Coronavirus 2
SPSS	Statistical Package for the Social Sciences
SD	Standard deviation
CPZ	Chlorpromazine
OAP	Oral antipsychotic
LAI	Long-acting injectable antipsychotic
OLZ	Olanzapine
RIS	Risperidone
ARI	Aripiprazole
QUE	Quetiapine
HAL	Haloperidol
FLX	Flupenthixol
ZUC	Zuclopenthixol
CLZ	Clozapine
BZD	Benzodiazepine
HAP	Hospital-acquired pneumonia
SMI	Severe mental illness
UK	United Kingdom
MECT	Modified electroconvulsive therapy
COPD	Chronic obstructive pulmonary disease
CYP2C19	Cytochrome P450 2C19
CYP1A2	Cytochrome P450 1A2
